# The role of sociodemographic, psychosocial, and behavioral factors in the use of preventive healthcare services in children and adolescents: results of the KiGGS Wave 2 study

**DOI:** 10.1186/s12887-024-04650-0

**Published:** 2024-02-28

**Authors:** Philip Bammert, Wiebke Schüttig, Iryna Iashchenko, Jacob Spallek, Petra Rattay, Sven Schneider, Matthias Richter, Claudia R Pischke, Nico Dragano, Leonie Sundmacher

**Affiliations:** 1 Chair of Health Economics, Department of Sports and Health Sciences, Technical University of Munich, Munich, Germany; 2 Department of Public Health, Brandenburg University of Technology Cottbus-Senftenberg, Senftenberg, Germany; 3 Lausitz Center for Digital Public Health, Brandenburg University of Technology Cottbus-Senftenberg, Senftenberg, Germany; 4 Department of Epidemiology and Health Monitoring, Robert Koch Institute, Berlin, Germany; 5 Center for Preventive Medicine and Digital Health (CPD), Division of General Medicine, Medical Faculty Mannheim, Heidelberg University, Mannheim, Germany; 6 Department of Sport and Health Sciences, Technical University of Munich, Munich, Germany; 7 Institute of Medical Sociology, Centre for Health and Society, University Hospital and Medical Faculty, University of Dusseldorf, Dusseldorf, Germany

**Keywords:** Healthcare use, Prevention, Inequity, Pediatrics, Vaccination

## Abstract

**Background:**

In Germany, various preventive services are offered to children and adolescents. These include regular standardized examinations (so called U/J examinations) and several vaccinations. Although strongly recommended, most of them are not mandatory. Our aim is to identify factors associated with the use of U/J examinations and vaccination against diphtheria, hepatitis B, Hib, pertussis, polio, and tetanus. While previous research has focused on sociodemographic factors, we also include socioeconomic, behavioral, and psychosocial factors.

**Methods:**

We analyzed cross-sectional data from 15,023 participants (aged 0–17 years) of the nationwide representative KiGGS Wave 2 Survey. Participation in U/J examinations was assessed using a questionnaire, filled out by participants and/or their parents. Information on vaccination status was drawn from the participants’ vaccination booklets. To identify relevant determinants for the use of preventive examinations and vaccinations, unadjusted and adjusted logistic regression models were employed with up to 16 different independent variables.

**Results:**

Various independent variables showed an association with the use of preventive services. Higher socioeconomic status, absence of migration background, and lower household size were associated with significantly higher utilization of U examinations. Parents’ marital status, area of residence, behavioral and psychosocial factors yielded insignificant results for most U/J examinations. Higher vaccination rates were found for children with no migration background, with residence in eastern Germany, lower household size, and with married parents.

**Conclusion:**

This study attempted to depict the influence of sociodemographic, psychosocial, and behavioral factors on the use of several preventive services. Our results indicate that predominantly sociodemographic variables influence the use of preventive services. Further efforts should be made to investigate the interplay of different determinants of healthcare use in children and adolescents.

**Supplementary Information:**

The online version contains supplementary material available at 10.1186/s12887-024-04650-0.

## Background

Equity in access to healthcare is a key premise for achieving equity in health and longevity [1, 2]. To evaluate equity in access, healthcare use serves as an important measure [3]. According to Andersen, healthcare use can be described as the realized and ‘effective access’ and is determined by need as well as predisposing and enabling factors. In contrast to other models related to healthcare use and health behaviors, in the Andersen model, health behaviors are regarded as factors that lead to healthcare utilization. It is thus suitable for the conceptualization of mechanisms of action in studies in the areas of social epidemiology and health services research [3].

Many high income countries, although often having high expenditures for health and offering a high level of insurance coverage for the population, still show inequities in health outcomes and the use of healthcare services [4–6]. Focusing on preventive measures, the existing evidence shows pro-well-off and pro-educated inequities in the vast majority of EU countries with regard to the use of various preventive measures [7]. Furthermore, poor and less educated people seem to have a higher probability of using preventive services late [8, 9]. Preventive measures here include services such as screenings and health examinations as well as vaccinations. This paper will focus on the use of childhood vaccinations and regular preventive health examinations in young people, the so-called U examinations and J examinations, in Germany.

Recommendations for vaccinations are given by the Standing Vaccination Committee. The so-called vaccination calendar, a booklet handed out to parents at the birth of their children, comprises recommendations for vaccination against childhood diseases, including tetanus, diphtheria, pertussis (whooping cough), Haemophilus influenzae type b (Hib), polio, and hepatitis B. The basic immunization for all these can be achieved with three inoculations of the respective vaccine [10].

It is further recommended that vaccination prophylaxis is conducted alongside the U examinations. These are standardized preventive measures recommended for children in Germany. In total, the U examinations consist of 12 separate examinations (U1–U11 + U7a), which are provided to children by pediatricians and general practitioners from birth to the age of 10 years. Additionally, two examinations are directed at adolescents. These are the J1 examination for adolescents aged 12–14 years and the J2 examination, which is recommended for adolescents aged 16–17 years. Of the total of 14 examinations, 11 are reimbursed by the social health insurances, whereas the U10, U11 and J2 examinations are only reimbursed by some sickness funds. Although strongly recommended, U/J examinations are mainly offered on a voluntary basis, the same as for vaccinations against diphtheria, hepatitis B, Hib, pertussis, polio, and tetanus. The exceptions here are three of the 16 German federal states, namely Bavaria, Baden-Württemberg, and Hesse, where U1–U9 examinations are mandatory. Also participation in the U examinations is sometimes a necessity in the school entry examination [11, 12]. Both the vaccinations and U/J examinations are free for every child and adolescent in Germany.

The contents and respective time periods for conducting the U/J examinations are constantly examined and republished in national legislation. Whereas each examination has its own focal points, their general aim is to insure healthy development of the child and early detection of diseases [12, 13]. An overview of the focal points and time periods of each U/J examination can be found in the Appendix.

Use of healthcare services is influenced by a variety of factors. As explained by Andersen [3], this includes predisposing characteristics and enabling factors. Predisposing characteristics refer to psychosocial factors that influence the decision-making process of an individual’s behavior. Enabling factors relate to resources that might be necessary to access care, such as the ability to pay [3]. This analysis will focus on sociodemographic, as well as psychosocial and behavioral factors as independent variables. The influence of socioeconomic status (SES) on the use of preventive care has already been covered in other studies [14–17]. According to the WHO Conceptual Framework on Social Determinants of Health [18], SES as a structural determinant operates through various intermediary determinants. These include psychosocial and behavioral factors, which in turn shape health outcomes. Psychosocial factors include, among others, psychosocial stressors and social support. Behavioral factors include, for example, nutrition, physical activity, and tobacco and alcohol consumption. As an addition to the existing literature, we therefore included factors representing these categories in the analysis [19–21].

The objective of this study is to comprehensively analyze possible influencing variables of uptake of several childhood vaccinations as well as the use of U/J examinations. We aim to answer the question: which sociodemographic, behavioral, and psychosocial factors are related to inequities in the use of preventive services in children?

## Methods

We analyzed cross-sectional data using unadjusted and adjusted logistic regression models to investigate the association of sociodemographic factors, behavioral, and psychosocial factors with participation in U/J examinations and vaccine uptake.

In the following, the dataset is described as well as how variables were operationalized and statistically investigated.

### Dataset

We used data from the population-based cross-sectional KiGGS Wave 2 study, which is the second follow-up to the German Health Interview and Examination Survey for Children and Adolescents (KiGGS) [22]. It comprised information for 15,023 children and adolescents aged 0–17 years with a primary residency status in Germany. Addresses to contact were drawn from official population registers of 167 cities and municipalities across Germany. Parents or legal guardians of all study participants took part in a structured interview. Additionally, from the age of 11, the participants were interviewed themselves. Furthermore, an examination routine was carried out. This yielded a rich dataset with information on a variety of subtopics. These were physical health, mental health, health-related behaviors such as dietary habits and physical activity, as well as healthcare and prevention use. Assessment of data took place between September 2014 and August 2017. A detailed description of the data that were assessed for KiGGS Wave 2 can be found elsewhere [23, 24]. For our analysis, the dataset was reduced to all participants with available data on the respective study variables. Thus, it has to be noted that the number of observations decreases with each U/J examination, because of the number of eligible participants.

### Dependent variables

To assess vaccination status, information was drawn from the children’s and adolescents’ vaccination booklets. This booklet is an official document to verify completed vaccinations and other associated measures. In this analysis, we differentiate between “fully vaccinated” and “not or insufficiently vaccinated” (i.e., received less than the recommended number of doses) participants. For diphtheria, Hib, hepatitis B, pertussis, polio, and tetanus, three injections are necessary to achieve “fully vaccinated” status.

Whether a child has attended a U examination was assessed via questionnaire, asking their parents, “Which U-preventive examinations did your child engage in?”. In the dataset, each variable regarding U or J examinations was depicted as a dichotomous indicator answered by either yes or no. Summing up vaccination and U/J examination variables results in 20 binary outcome variables, which were analyzed.

### Independent variables

#### Sociodemographic variables

A variety of sociodemographic variables was extracted from the dataset. Many of them have been shown to be associated with service use in other studies [25–27]. These were age, gender, area of residence (eastern or western Germany), parents’ marital status, migration background, household size, and socioeconomic status (SES), which accounted for the parents’ income, education, and occupational status. The definition of the migration background in KiGGS is based on the country of birth of both the child and the parents, as well as on the citizenship of the parents. Thus, a two-sided migration background is given if both parents were not born in Germany or do not have German citizenship. If the child and at least one of its parents were born abroad, this is also defined as a two-sided migration background. A one-sided migration background is given if one parent was born abroad or does not have German citizenship [28]. Instead of using the domains of income, education, and occupational status separately, we decided to apply the prefabricated SES index in accordance with other studies that are based on the KiGGS Wave 2 dataset [29]. As a short explanation, parents were asked for their income in euros, their highest scholarly and occupational qualification adapted to the CASMIN classification (Comparative Analysis of Social Mobility in Industrial Nations) [30], and their occupational status, which was evaluated using the criteria of ISEI (International Socio-Economic Index of Occupational Status) [31]. Each of the three domains then received a score between 1 and 7, according to prespecified thresholds. The composite index was then calculated with each domain having the same weight [32]. The index value can be categorized into low [1–7], medium [8–14], and high SES [15–21]. This categorization was used as an independent variable in this analysis. The exact composition of the index and all corresponding threshold values can be found elsewhere [32].

#### Behavioral variables

We added the following behavioral variables relating to the participants themselves: number of days with equal to or more than 60 min of physical activity per week, fast-food consumption per week, and for participants aged 11 years or older, an indicator for whether they have ever consumed alcohol as well as an indicator on whether they have ever smoked. Additionally, parental smoking status was assessed. All these variables were derived from questionnaire results. Participants aged 11 years or older filled in the questionnaires themselves, whereas it was their parents or a legal guardian who responded for younger participants [23]. Fast-food consumption was indicated by a variable that indicated how often certain meals are consumed per week, including burgers, fries, pizza, kebabs, and currywurst. Several studies showed that increasing fast-food consumption is significantly associated with poorer dietary quality, higher energy intakes, and an elevated risk of obesity [33, 34].

#### Psychosocial variables

The following psychosocial variables were assessed: family cohesion, personal resources, self-efficacy, and social support. All these variables were obtained using specific questionnaires, answered by the participants themselves, but only for those aged 11 years or older. The exception is family cohesion, which was assessed for participants aged 3 years or older. Family cohesion, which can be defined as the emotional bonding of family members while accounting for the degree of individual autonomy a person experiences [35], was assessed by a shortened version of the so-called Family Climate Scale, originally developed by Schneewind, Beckmann, and Hecht-Jackl [36]. Personal resources were measured by a subset of the Bern Wellbeing Questionnaire [37] and a subset of the SOC – Sense of Coherence Scale [38]. Self-efficacy, which is defined as the trust of a person in their own capability to handle difficult situations, according to Bandura [39], was evaluated using the Scale for Measuring General Self-efficacy Beliefs [40]. The variable of social support was assessed on the SSS – Social Support Scale [41]. It indicates how well psychosocial needs for affiliation and recognition are met [42]. The four variables were transformed into scores ranging from 0 to 100, with higher scores indicating better psychosocial resources.

### Statistical approach

First, sample characteristics were computed for the whole sample. As a core method, binomial logistic regression was used. Key assumptions for the correct usage of this method have been tested in advance. The included variables were checked for multicollinearity using the variance inflation factor. Similarly, sample size calculations were conducted using G*Power to insure adequacy of the sample size for each regression model. For each outcome, an unadjusted and an adjusted regression model including all variables assessed were set up. Variables, which were only assessed for participants at a certain minimum age, were not considered for outcomes that are normally provided at an earlier age. Thus, for all vaccinations, fast-food consumption, physical activity, smoking, and drinking behavior, as well as all psychosocial variables, were not included in the regression models. For the same reason, regarding U examinations, physical activity, fast-food consumption, and the indicator for family cohesion occurred first in the models for U8. Other psychosocial variables as well as smoking and drinking behavior were used only for J1 and J2. In total, the results from a total of 20 regression models are reported. Results are reported as adjusted odds ratios (aOR). All statistical procedures were conducted with R. The calculations were carried out using a weighting factor that corrected for deviations within the sample from the population structure with regard to age, sex, region, nationality, and parents’ education level. Owing to the complex survey structure and the clustering of sample points, the R-package “survey” was used [24].

## Results

Table 1 shows the distribution of the main sample characteristics. Considering the sociodemographic composition, the largest proportion of participants came from families with medium SES, the second largest share came from families with low SES, and the smallest share from families with high SES, with 59.9%, 20.1%, and 20.0% respectively. Most parents were married, and mean household size was 4. Approximately 70% of participants had no migration background (71.2%), whereas the other 30% had a one-sided or two-sided migration background (11.8% and 17.0% respectively).

**Table 1:** Sample characteristics

Descriptive summary		
Variable (n)		Mean (standard error) / %
Gender (15,023)	female	50.2%
	male	49.8%
Age (15,023)		8.8 (0.04)
SES (14,790)	low	20.1%
	medium	59.9%
	high	20.0%
Parents’ marital status (14,677)	single	12.6%
	married	76.2%
	other	11.2%
Household size (14,659)		4.1 (0.02)
Migration background (14,851)	none	71.2%
	one-sided	11.8%
	two-sided	17.0%
Area of residence (15,023)	western Germany	81.9%
	eastern Germany	18.1%
Physical activity: active days per week (12,981)		4.5 (0.03)
Fast-food items per week (12,909)		1.8 (0.02)
Ever consumed alcohol (11 years or older) (6,141)	yes	51.0%
	no	49.0%
Ever smoked (11 years or older) (5,747)	yes	6.6%
	no	93.4%
Parents’ smoking status (14,154)	both	13.1%
	one	24.5%
	none	62.4%

Table 2 shows overall participation rates in examinations and vaccinations. Participation in the U examinations is continuously very high, except for U10 and U11. These are the only two U examinations in the set that are not reimbursed by all sickness funds. There is an overall trend of decreasing participation for later examinations. For adolescents, it is noticeable that, compared with J1, participation in J2 is much lower, with only 37% of adolescents taking part. Again, it should be noted that J1 is reimbursed by all sickness funds mandatorily, which is not the case for J2. Additionally, it should be considered that the older a participant is, the stronger is his or her own influence on healthcare utilization, compared with the influence of the parent or legal guardian. Looking at vaccinations, participation rates are high throughout all of the six vaccinations, with five of them having a participation rate higher than 94%.

**Table 2:** Participation rates for use of U-preventive examinations and vaccinations

Examination	n	%
U1	14,017	98.7%
U2	13,968	98.7%
U3	13,964	98.7%
U4	13,865	98.4%
U5	13,602	98.3%
U6	13,266	98.3%
U7	12,773	98.0%
U7a	11,679	91.7%
U8	11,536	96.9%
U9	10,358	96.9%
U10	8,103	80.3%
U11	6,383	73.6%
J1	3,537	66.7%
J2	846	37.0%
Vaccination	n	%
Diphtheria	3,238	96.2%
Hepatitis B	3,238	84.3%
Hib	3,238	92.5%
Pertussis	3,238	94.7%
Polio	3,238	94.2%
Tetanus	3,238	96.5%

For the sake of clarity, the following tables include adjusted odds ratios and confidence intervals only for a selection of all outcomes. These are the vaccinations against pertussis, polio, and tetanus as well as the U1, U10, and J1 examination. Table 3 gives an overview of significant associations between the analyzed variables and respective outcomes. Complete tables of all unadjusted and adjusted regression models for each outcome can be found in the Appendix.

**Table 3:** Overview of significant influencing factors for use of each vaccination and U/J examination

	Variable	Vaccination use		U/J examination use	
		Positive relationship	Negative relationship	Positive relationship	Negative relationship
Socioeconomic & Demographic	Gender	–	–	–	–
	SES score	Diphtheria, Polio, Tetanus	–	U1, U2, U3, U4, U5, U6, U7, U8	J2
	One-sided migration background (yes vs. no)	Diphtheria, Hib, Polio, Tetanus	–	–	–
	Two-sided migration background (yes vs. no)	–	Diphtheria, Hib, Polio, Tetanus	–	U1, U2, U3, U4, U5, U6, U7, U7a, U8, U9
	Area of residence (west vs. east)	–	Hepatitis B, Pertussis	U7a, U8	–
	Household size	–	Diphtheria, Hepatitis B, Pertussis, Polio, Tetanus	J2	–
	Parents’ marital status (married vs. single)	Diphtheria, Polio, Tetanus	–	–	–
Psycho–social	Family cohesion	n.a.	n.a.	–	–
	Personal resources	n.a.	n.a.	–	–
	Self-efficacy	n.a.	n.a.	–	–
	Social support	n.a.	n.a.	–	–
	Parents’ smoking status (one vs. none)	–	–	–	–
Behavioral	Parents’ smoking status (both vs. none)	Diphtheria, Hepatitis B, Pertussis, Tetanus	–	–	–
	Physical activity: active days per week	n.a.	n.a.	U10	–
	Fast-food consumption	n.a.	n.a.	–	U8, U9
	Ever consumed alcohol	n.a.	n.a.	–	–
	Ever smoked	n.a.	n.a.	–	–

Positive relationship = increase in variable leads to higher odds of service use; variable SES = socioeconomic status; n.a. = not applicable – variable was not assessed for the respective outcome; household size measured in number of inhabitants; family cohesion, personal resources, self-efficacy, and social support measured in scales from 0 to 100

Table 4 shows the results of unadjusted and adjusted logistic regressions for the selected vaccinations. Various independent variables turned out to be significant in the analysis. Medium SES is only significant for the uptake of diphtheria and tetanus immunization, when comparing medium SES with low SES. Living in a family of high SES compared with low SES is significant for the tetanus vaccination. Migration background shows significant influence in three of the vaccinations, diphtheria, Hib, and tetanus. For children with a two-sided migration background, the odds of a completed vaccination against Hib and tetanus are significantly lower compared with children with no migration background. Conversely though, for diphtheria, Hib, and tetanus, the odds seem to be significantly higher when the participant has a one-sided migration background. The area of residence as well as the parents’ marital status do not seem to influence the odds for vaccine uptake, whereas for participants with a larger household size, the odds are significantly lower for vaccination against polio. Of the behavioral variables, only the parents’ smoking status was assessed for the vaccination-related outcomes. The odds for full vaccination status are significantly higher for diphtheria, hepatitis B, pertussis, and tetanus when both parents smoke compared with none.

**Table 4:** Adjusted and unadjusted regression models for vaccination uptake against pertussis, polio, and tetanus

		Pertussis (*n* = 3,238)				Polio (*n* = 3,238)				Tetanus (*n* = 3,238)			
		unadjusted		adjusted		unadjusted		adjusted		unadjusted		adjusted	
		aOR	CI	aOR	CI	aOR	CI	aOR	CI	aOR	CI	aOR	CI
Age		0.99	0.94–1.06	0.94	0.92–0.96	1.00	0.95–1.05	0.98	0.95–1.02	1.06	0.98–1.14	1.05	1.00–1.10
Gender	female	1		1		1		1		1		1	
	male	1.15	0.77–1.71	0.95	0.78–1.17	0.96	0.64–1.47	0.95	0.68–1.33	1.19	0.71–2.00	1.07	0.69–1.68
SES score	low	1		1		1		1		1		1	
	medium	2.36	1.33–4.21	1.16	0.87–1.53	2.24	1.32–3.81	1.72	1.13–2.60	3.12	1.58–6.21	2.91	1.71–4.97
	high	1.57	0.84–2.98	1.08	0.76–1.54	2.02	1.14–3.60	1.26	0.74–2.18	3.14	1.43–6.91	2.2	1.09–4.64
Migration background	none	1		1		1		1		1		1	
	one-sided	3.17	1.31–7.70	1.15	0.81–1.68	2.12	0.79–5.73	2.20	1.05–5.59	5.11	1.54–16.88	4.77	1.31–38.23
	two-sided	0.39	0.23–0.67	1.00	0.76–1.32	0.41	0.25–0.68	0.62	0.42–0.93	0.29	0.25–0.55	0.45	0.27–0.75
Area of residence	east	1		1		1		1		1		1	
	west	0.96	0.45–2.05	0.70	0.52–0.94	1.11	0.56–2.20	1.17	0.74–1.81	1.35	0.57–3.25	1.56	0.87–2.68
Household size		0.71	0.57–0.90	0.89	0.81–0.97	0.76	0.60–0.97	0.83	0.74–0.93	0.68	0.52–0.89	0.86	0.75–0.99
Parents’ marital status	single	1		1		1		1		1		1	
	married	1.38	0.70–2.74	0.99	0.99–2.14	1.55	0.81–2.95	1.87	1.16–3.39	1.73	0.74–4.08	2.85	1.16–4.18
Parents’ smoking status	none	1		1		1		1		1		1	
	one parent	1.26	0.72–2.20	1.19	0.93–1.52	1.02	0.60–1.76	1.05	0.72–1.55	1.09	0.52–2.27	1.18	0.73–1.94
	both parents	2.07	1.04–4.17	1.73	1.24–2.47	1.42	0.75–2.70	1.57	0.93–2.79	2.27	0.90–5.74	3.57	1.60–9.48

aOR = adjusted odds ratio; CI = confidence interval; adjusted models included all listed variables adjusted for each other; age measured in years; household size measured in number of inhabitants

Table 5 shows the results of unadjusted and adjusted regression models for the uptake of U1, U10, and J1. SES is a significant independent variable in investigating the uptake of U1–U8 as well as J2. Living in a family with medium SES compared with low SES significantly raises the odds of completion for eight of the 14 examinations, whereas living in a family with high SES compared with low SES does so for four of them. For J2, participants with high SES are significantly less likely to have taken part in the examination compared with participants with low SES. Migration background is a significant independent variable when investigating 10 of the examinations. When comparing a one-sided migration background to none, the odds do not change significantly between the groups, whereas when comparing a two-sided migration background to none, a two-sided migration background significantly lowers the odds for the completion of 10 examinations. Living in western Germany is a positive predictor for U7a and U8 only. Other than that, area of residence seems not to influence vaccination uptake. Increasing household size reduces the odds of completion significantly for two of the 14 outcomes, whereas children with married parents have lower odds for four of them compared with children with unmarried parents. Looking at the behavioral variables, only a few significant results were computed. Children with higher fast-food consumption per week have significantly lower odds for participation in two of the U/J examinations. Children with one smoking parent have significantly lower odds for participation in U7a. The number of sufficiently physically active days per week is not associated with any of the U/J examinations, as well as previous consumption of alcohol or tobacco.

**Table 5:** Adjusted and unadjusted regression models for U1, U10, and J1 examination use

			U1 (*n* = 14,017)				U10 (*n* = 8,103)				J1 (*n* = 3,537)			
			unadjusted		adjusted		unadjusted		adjusted		unadjusted		adjusted	
			aOR	CI	aOR	CI	aOR	CI	aOR	CI	aOR	CI	aOR	CI
Age			0.97	0.92–1.03	0.98	0.93–1.04	1.01	0.99–1.05	1.03	1.00–1.07	1.34	1.26–1.42	1.36	1.25–1.48
Gender		female	1		1		1		1		1		1	
		male	1.25	0.77–2.07	1.45	0.80–2.64	1.15	1.00–1.32	1.12	0.96–1.31	1.09	0.90–1.34	0.97	0.76–1.24
SES score		low	1		1		1		1		1		1	
		medium	7.32	4.13–13.01	2.92	1.60–5.35	1.39	1.13–1.72	1.13	0.89–1.45	1.39	1.10–1.77	1.34	0.96–1.88
		high	10.37	5.11–21.04	2.39	1.06–5.39	1.08	0.87–1.35	0.87	0.66–1.13	1.13	0.85–1.50	1.06	0.71–1.57
Migration background		none	1		1		1		1		1		1	
		one-sided	1.07	3.26	1.39	0.35–5.59	1.13	0.88–1.48	1.15	0.86–1.53	0.91	0.68–1.22	1.02	0.72–1.44
		two-sided	0.01	0.01–0.03	0.02	0.01–0.04	0.69	0.53–0.89	0.83	0.63–1.08	0.76	0.57–1.03	1.05	0.73–1.52
Area of residence		east	1		1		1		1		1		1	
		west	0.53	0.27–1.04	0.92	0.35–2.41	0.96	0.80–1.16	0.97	0.79–1.19	0.85	0.67–1.08	0.84	0.63–1.12
Household size			0.70	0.58–0.84	1.02	0.80–1.29	0.88	0.80–0.96	0.90	0.83–0.99	0.97	0.88–1.07	1.01	0.89–1.15
Parents’ marital status		single	1		1		1		1		1		1	
		married	0.35	0.13–0.95	0.35	0.07–1.82	1.09	0.82–1.45	1.19	0.85–1.67	1.29	0.88–1.89	1.32	0.80–2.16
Parents’ smoking status		none	1		1		1		1		1		1	
		one parent	1.03	0.53–2.01	1.31	0.64–2.67	1.12	0.92–1.36	1.07	0.86–1.33	1.00	1.00–1.01	0.98	0.75–1.28
		both parents	1.81	0.68–4.82	2.25	0.78–6.50	1.05	0.84–1.30	1.01	0.80–1.26	1.00	1.00–1.01	1.01	0.71–1.45
Physical activity: active days per week			–	–	–	–	1.03	0.99–1.08	1.06	1.01–1.11	1.00	0.99–1.01	1.01	0.91–1.12
Fast-food consumption			–	–	–	–	0.96	0.92–1.01	0.98	0.93–1.04	1.00	1.00–1.01	0.97	0.88–1.06
Family cohesion			–	–	–	–	–	–	–	–	0.99	0.79–1.24	1.00	1.00–1.01
Personal resources			–	–	–	–	–	–	–	–	0.93	0.70–1.23	1.01	1.00–1.02
Self-efficacy			–	–	–	–	–	–	–	–	0.97	0.92–1.02	0.99	0.98–1.01
Social support			–	–	–	–	–	–	–	–	0.98	0.93–1.02	1.01	1.00–1.01
Ever consumed alcohol	no		–	–	–	–	–	–	–	–	1		1	
	yes		–	–	–	–	–	–	–	–	1.98	1.67–2.34	1.34	0.62–2.90
Ever smoked	no		–	–	–	–	–	–	–	–	1		1	
	yes		–	–	–	–	–	–	–	–	1.34	0.89–2.02	1.03	0.54–1.99

aOR = adjusted odds ratio; CI = confidence interval; adjusted models included all listed variables adjusted for each other; age measured in years; household size measured in number of inhabitants; family cohesion, personal resources, self-efficacy, and social support measured in scales from 0 to 100

## Discussion

Existing social inequalities in the use of preventive examinations, meaning that children in families with a lower SES tend to use them less, have been shown in previous studies and local health reports [43–48]. The majority of comparable studies are thus in line with the results of this analysis. Nevertheless, there are also studies that came to the conclusion that SES is not significantly associated with the use of U examinations [49, 50]. This discrepancy could be explained by differences in study design, statistical methodology, and sampling. For example, other reports used survey data or restricted their analysis to descriptive statistics. Our finding of a strong association of migration background with prevention use can be found in all comparable studies that accounted for the variable [43, 47, 50–52]. Reasons for this are manifold. First, it should be mentioned that, in this study, children were included in the analysis irrespective of their country of birth. In many cases, children born outside Germany were still abroad at the time when early U examinations would have taken place [53]. This is especially likely when the participant has a two-sided migration background. Other than that, it is to be assumed that, for families with a migration background, lack of familiarity with the healthcare system or the ability to speak German may prevent them from using all of the services offered. Furthermore, the effect of area of residence was examined in a different study based on data from the KiGGS baseline. Interestingly, the study found that living in western Germany acts as a positive predictor only starting from the U6 examination [43]. We assume that this disparity comes from differences in regional promotion strategies for the examinations and judicial differences in the established reporting systems [54, 55]. For some outcomes, age was a significant predictor. However, this can be seen as a logical byproduct of the analysis because the older a child or adolescent is, the higher the chance of having participated in a measure available for a certain age range.

Comparing our findings on determinants of vaccination status with other studies conducted in Germany and other developed countries, we detect some similarities but also differences. SES is considered to be a significant predictor for vaccination uptake in many studies [56, 57]. A systematic review including studies from 39 developed countries came to the same conclusion [58]. Nevertheless, the evidence is unclear, as there are also studies reporting an insignificant effect of SES [49, 59, 60]. In our results, SES was only significant for two of the vaccinations. Furthermore, the literature suggests contrary results regarding the influence of migration background. Although some studies suggest that having a migration background significantly lowers the chances of being fully vaccinated against some of the selected diseases [49, 56, 60], others indicate that children with a one- or twofold migration background could even show higher vaccination rates [47, 57]. Our findings partly agree with both takes as, in our dataset, compared with children with no migration background, those with a two-sided migration background are less likely to be vaccinated against Hib and tetanus, whereas those with a one-sided migration background are more likely to be vaccinated against diphtheria, Hib, and tetanus. We assume that the country of origin and its specific vaccination recommendations have an effect on the likelihood of vaccination. The negative influence of household size on vaccination uptake appears frequently in the literature and is aligned with the presented findings. Larger household size is significantly associated with a lower likelihood of completeness of vaccinations that are recommended in early childhood [47, 49, 57, 61]. A possible explanation could be that finding childcare for the children’s siblings during physician appointments can pose a barrier. Investigating psychosocial variables, systematic reviews suggest that, among others, negative beliefs toward immunization, fear of side-effects, and trust in the healthcare profession are further relevant factors in this context [58, 62].

The results of our analysis come with some limitations, arising from the way the dependent and independent variables were measured and operationalized. For instance, the vaccination-related outcomes were dichotomous and only differentiated between complete immunization and insufficient immunization. An in-depth analysis could further differentiate between the numbers of injections that were received. Furthermore, even though a larger number of variables were drawn from the dataset, only a limited number could be used for examinations and vaccinations, which occur in the early stages of life. As most of the variables were assessed via questionnaire, the possible existence of a recall bias must be considered. Also, this analysis makes no claim to complete inclusion of all possible influencing factors. This analysis focused on socioeconomic, psychosocial, and behavioral variables; however, other factors, such as rurality or health status, could further influence healthcare utilization. Last, it should be acknowledged that there are multiple ways to depict SES. For this study, we chose to use an indicator that combined and summarized the dimensions of income, education, and occupation to a numerical value. We chose this composite index because it enables a holistic operationalization of SES as a multidimensional construct. This operationalization is also the most widely used method in comparable study designs [32]. However, this could lead to limitations regarding comparability with studies using other measures of SES. Furthermore, it must be mentioned that the cross-sectional data as well as the large number of included variables do not allow us to draw causal conclusions.

As strengths of this study, three aspects should be mentioned: First, the data used come from the KiGGS Wave 2 study [23]. Hence, the analysis is based on reliable and high-quality data. Second, we were able to use a large variety of independent variables. To our knowledge, this study is the first to combine sociodemographic, psychosocial, and behavioral factors in the context of the uptake of U/J examinations and childhood vaccinations. Third, we used logistic regression as the main statistical method, whereas other studies examining a comparable research topic often relied solely on stratified participation rates.

## Conclusions

To sum up, several sociodemographic variables seem to be associated with the use of recommended U/J examinations and vaccinations. These include socioeconomic position, migration background, area of residence, and household size. These results point to several population groups that should be targeted with prevention-promoting strategies. Additionally, the results hint that behavioral and psychosocial factors might also play a role in the use of U/J examinations and vaccinations. Further research should test the relevance of these factors on inequity in healthcare use in greater depth to possibly identify starting points for health interventions.

### Electronic supplementary material

Below is the link to the electronic supplementary material.

Supplementary Material 1

Supplementary Material 2

Supplementary Material 3

## Data Availability

The dataset analyzed during the current study is not publicly available as it is owned by the Robert Koch Institute, but is available from the Robert Koch Institute on reasonable request.
