# Supplementary material for: The role of sociodemographic, psychosocial, and behavioral factors in the use of preventive healthcare services in children and adolescents: results of the KiGGS Wave 2 study
Source: BMC Pediatr. 2024 Feb 28;24:146. doi: 10.1186/s12887-024-04650-0 (PMC10900680; doi:10.1186/s12887-024-04650-0)
Supplement: Supplementary file 1 — Supplementary Material 1 [file 12887_2024_4650_MOESM1_ESM.docx]

A1: Contents of U-/J-examinations

|  | Age | Focus Points |
| --- | --- | --- |
| U1 | After birth | Detect life-threatening complications, birth trauma and malformations, capture pre-, peri- and postnatal risk factors, determine further care |
| U2 | 3.-10. day of life | Detect innate diseases and pathologic jaundice, screenings for heart defects, cystic fibrosis and hearing ability, parent child interaction |
| U3 | 4.-5. week of life | Detect developmental abnormalities (motor skills, speech, cognition, emotional competence, body) and pathologic jaundice, hip joint sonography, parent child interaction, vaccination counselling |
| U4 | 3.-4. month of life | Detect developmental abnormalities (motor skills, speech, cognition, emotional competence, body), vaccination counselling, newborn hearing screening, parent child interaction |
| U5 | 6.-7. month of life | Detect developmental abnormalities (motor skills, speech, cognition, emotional competence, body) and visual impairments, vaccination counselling, newborn hearing screening, parent child interaction |
| U6 | 10.-12. month of life | Detect developmental abnormalities (motor skills, speech, cognition, emotional competence, body) and visual impairments, vaccination counselling, parent child interaction |
| U7 | 21.-24. month of life | Detect developmental abnormalities (motor skills, speech, cognition, emotional competence, body) and visual impairments, vaccination counselling, parent child interaction |
| U7a | 34.-36. month of life | Detect developmental abnormalities (motor skills, speech, cognition, emotional competence, body) and visual impairments, vaccination counselling, parent child interaction |
| U8 | 46.-48. month of life | Detect developmental abnormalities (motor skills, speech, cognition, emotional competence, body) and visual impairments, examine hearing ability, vaccination counselling, parent child interaction |
| U9 | 60.-64 month of life | Detect developmental abnormalities (motor skills, speech, cognition, emotional competence, body) and visual impairments, parent child interaction |
| U10 | ages 7-8 | Detect developmental abnormalities (motor skills, speech, cognition, emotional competence, body) school performance disorders, tooth anomalies and behavioral disorders, vaccination counselling |
| U11 | ages 9-10 | Detect school performance disorders, tooth anomalies and behavioral disorders, vaccination counselling, assess media consumption |
| J1 | ages 12-14 | Examine overall health, growth development, state of puberty and school performance, vaccination counselling, detect psychological abnormalities, assess health risking behaviors (smoking, alcohol, drugs) |
| J2 | ages 16-17 | Assess behavioral and sexuality disorders, detect postural disorders and goiter, diabetes prevention, career counselling |
