# Supplementary material for: The role of sociodemographic, psychosocial, and behavioral factors in the use of preventive healthcare services in children and adolescents: results of the KiGGS Wave 2 study
Source: BMC Pediatr. 2024 Feb 28;24:146. doi: 10.1186/s12887-024-04650-0 (PMC10900680; doi:10.1186/s12887-024-04650-0)
Supplement: Supplementary file 2 — Supplementary Material 2 [file 12887_2024_4650_MOESM2_ESM.docx]

*Table B1: Unadjusted regression models for vaccination uptake*

|  | | Diphtheria | | Hepatitis B | | Hib | | Pertussis | | Polio | | Tetanus | |
| --- | --- | --- | --- | --- | --- | --- | --- | --- | --- | --- | --- | --- | --- |
|  |  | n = 3,238 | | n = 3,238 | | n = 3,238 | | n = 3,238 | | n = 3,238 | | n = 3,238 | |
|  |  | aOR | CI | aOR | CI | aOR | CI | aOR | CI | aOR | CI | aOR | CI |
| Age |  | 1.05 | 0.98-1.13 | 0.94 | 0.92-0.97 | 0.93 | 0.89-0.99 | 0.99 | 0.94-1.06 | 1.00 | 0.95-1.05 | 1.06 | 0.98-1.14 |
| Gender | female | 1 |  | 1 |  | 1 |  | 1 |  | 1 |  | 1 |  |
|  | male | 1.16 | 0.71-1.91 | 0.96 | 0.75-1.24 | 0.79 | 0.57-1.08 | 1.15 | 0.77-1.71 | 0.96 | 0.64-1.47 | 1.19 | 0.71-2.00 |
| SES score | low | 1 |  | 1 |  | 1 |  | 1 |  | 1 |  | 1 |  |
|  | medium | 2.79 | 1.43-5.46 | 1.11 | 0.75-1.65 | 1.82 | 1.15-2.87 | 2.36 | 1.33-4.21 | 2.24 | 1.32-3.81 | 3.12 | 1.58-6.21 |
|  | high | 2.50 | 1.14-5.49 | 1.06 | 0.73-1.56 | 1.61 | 0.97-2.69 | 1.57 | 0.84-2.98 | 2.02 | 1.14-3.60 | 3.14 | 1.43-6.91 |
| Migration background | none | 1 |  | 1 |  | 1 |  | 1 |  | 1 |  | 1 |  |
|  | one-sided | 3.74 | 1.26-11.04 | 1.17 | 0.73-1.90 | 2.19 | 1.10-4.38 | 3.17 | 1.31-7.70 | 2.12 | 0.79-5.73 | 5.11 | 1.54-16.88 |
|  | two-sided | 0.33 | 0.18-0.63 | 0.87 | 0.63-1.21 | 0.53 | 0.33-0.86 | 0.39 | 0.23-0.67 | 0.41 | 0.25-0.68 | 0.29 | 0.25-0.55 |
| Area of residence | east | 1 |  | 1 |  | 1 |  | 1 |  | 1 |  | 1 |  |
|  | west | 1.34 | 0.59-3.02 | 0.78 | 0.48-1.27 | 1.14 | 0.67-1.95 | 0.96 | 0.45-2.05 | 1.11 | 0.56-2.20 | 1.35 | 0.57-3.25 |
| Household size |  | 0.68 | 0.52-0.89 | 0.85 | 0.73-0.99 | 0.85 | 0.69-1.06 | 0.71 | 0.57-0.90 | 0.76 | 0.60-0.97 | 0.68 | 0.52-0.89 |
| Parents' marital status | single | 1 |  | 1 |  | 1 |  | 1 |  | 1 |  | 1 |  |
|  | married | 1.66 | 0.73-3.78 | 0.91 | 0.62-1.37 | 1.08 | 0.60-1.96 | 1.38 | 0.70-2.74 | 1.55 | 0.81-2.95 | 1.73 | 0.74-4.08 |
| Parents' smoking status | none | 1 |  | 1 |  | 1 |  | 1 |  | 1 |  | 1 |  |
|  | one parent | 1.06 | 0.53-2.11 | 1.20 | 0.86-1.68 | 0.96 | 0.62-1.49 | 1.26 | 0.72-2.20 | 1.02 | 0.60-1.76 | 1.09 | 0.52-2.27 |
|  | both parents | 2.17 | 0.91-5.20 | 1.79 | 1.21-2.67 | 1.39 | 0.73-2.69 | 2.07 | 1.04-4.17 | 1.42 | 0.75-2.70 | 2.27 | 0.90-5.74 |

*Table B2a: Unadjusted models for U-examination use, U1–U4*

|  | | U1 | | U2 | | U3 | | U4 | |
| --- | --- | --- | --- | --- | --- | --- | --- | --- | --- |
|  |  | n = 14,017 | | n = 13,968 | | n = 13,964 | | n = 13,865 | |
|  |  | aOR | CI | aOR | CI | aOR | CI | aOR | CI |
| Age |  | 0.97 | 0.92-1.03 | 0.98 | 0.77-2.07 | 0.98 | 0.93-1.04 | 0.97 | 0.92-1.03 |
| Gender | female | 1 |  | 1 |  | 1 |  | 1 |  |
|  | male | 1.25 | 0.77-2.07 | 1.28 | 0.79-2.10 | 1.19 | 0.75-1.91 | 1.35 | 0.86-2.16 |
| SES score | low | 1 |  | 1 |  | 1 |  | 1 |  |
|  | medium | 7.32 | 4.13-13.01 | 6.82 | 3.96-11.78 | 6.41 | 3.65-11.26 | 6.77 | 4.00-11.47 |
|  | high | 10.37 | 5.11-21.04 | 11.79 | 5.85-23.79 | 10.08 | 5.20-19.55 | 10.04 | 4.86-20.74 |
| Migration background | none | 1 |  | 1 |  | 1 |  | 1 |  |
|  | one-sided | 1.07 | 3.26 | 1.22 | 0.41-3.60 | 1.35 | 0.46-3.97 | 0.30 | 0.10-0.91 |
|  | two-sided | 0.01 | 0.01-0.03 | 0.01 | 0.01-0.03 | 0.02 | 0.01-0.03 | 0.03 | 0.01-0.05 |
| Area of residence | east | 1 |  | 1 |  | 1 |  | 1 |  |
|  | west | 0.53 | 0.27-1.04 | 0.73 | 0.36-1.48 | 0.63 | 0.35-1.15 | 0.49 | 0.28-0.88 |
| Household size |  | 0.70 | 0.58-0.84 | 0.72 | 0.60-0.85 | 0.72 | 0.60-0.86 | 0.69 | 0.58-0.82 |
| Parents' marital status | single | 1 |  | 1 |  | 1 |  | 1 |  |
|  | married | 0.35 | 0.13-0.95 | 0.51 | 0.21-1.22 | 0.54 | 0.23-1.31 | 0.91 | 0.38-2.16 |
| Parents' smoking status | none | 1 |  | 1 |  | 1 |  | 1 |  |
|  | one parent | 1.03 | 0.53-2.01 | 0.99 | 0.55-1.82 | 0.91 | 0.50-1.68 | 1.13 | 0.61-2.09 |
|  | both parents | 1.81 | 0.68-4.82 | 2.01 | 0.78-5.20 | 1.92 | 0.74-4.98 | 1.72 | 0.71-4.20 |

*Table B2b: Unadjusted regression models for U-examination use, U5–U7a*

|  | | U5 | | U6 | | U7 | | U7a | |
| --- | --- | --- | --- | --- | --- | --- | --- | --- | --- |
|  |  | n = 13,602 | | n = 13,266 | | n = 12,773 | | n = 11,679 | |
|  |  | aOR | CI | aOR | CI | aOR | CI | aOR | CI |
| Age |  | 0.96 | 0.92-1.02 | 0.92 | 0.87-0.98 | 0.94 | 0.89-0.99 | 0.84 | 0.82-0.86 |
| Gender | female | 1 |  | 1 |  | 1 |  | 1 |  |
|  | male | 1.23 | 0.79-1.93 | 1.10 | 0.68-1.77 | 1.05 | 0.69-1.61 | 1.03 | 0.86-1.24 |
| SES score | low | 1 |  | 1 |  | 1 |  | 1 |  |
|  | medium | 5.75 | 3.38-9.79 | 6.29 | 3.78-10.47 | 4.71 | 2.97-7.46 | 1.47 | 1.11-1.94 |
|  | high | 7.27 | 3.86-13.69 | 7.59 | 4.08-14.13 | 5.01 | 2.99-8.39 | 1.48 | 1.08-2.02 |
| Migration background | none | 1 |  | 1 |  | 1 |  | 1 |  |
|  | one-sided | 0.89 | 0.35-2.25 | 0.75 | 0.30-1.85 | 0.95 | 0.43-2.10 | 1.17 | 0.87-1.59 |
|  | two-sided | 0.04 | 0.02-0.06 | 0.04 | 0.03-0.05 | 0.06 | 0.04-0.09 | 0.47 | 0.37-0.60 |
| Area of residence | east | 1 |  | 1 |  | 1 |  | 1 |  |
|  | west | 0.68 | 0.41-1.11 | 0.94 | 0.56-1.56 | 1.12 | 0.77-1.63 | 1.05 | 0.86-1.28 |
| Household size |  | 0.71 | 0.60-0.84 | 0.71 | 0.58-0.84 | 0.75 | 0.64-0.88 | 0.88 | 0.80-0.97 |
| Parents' marital status | single | 1 |  | 1 |  | 1 |  | 1 |  |
|  | married | 0.82 | 0.33-2.01 | 0.88 | 0.35-2.23 | 1.03 | 0.58-1.86 | 0.83 | 0.57-1.20 |
| Parents' smoking status | none | 1 |  | 1 |  | 1 |  | 1 |  |
|  | one parent | 1.12 | 0.65-1.94 | 0.97 | 0.57-1.67 | 0.83 | 0.52-1.34 | 1.23 | 1.00-1.51 |
|  | both parents | 1.77 | 0.81-3.86 | 1.67 | 0.75-3.75 | 0.88 | 0.49-1.57 | 1.19 | 0.89-1.59 |

*Table B2c: Unadjusted regression models for U-examination use, U8-U11*

|  | | U8 | | U9 | | U10 | | U11 | |
| --- | --- | --- | --- | --- | --- | --- | --- | --- | --- |
|  |  | n = 11,536 | | n = 10,358 | | n = 8,103 | | n = 6,383 | |
|  |  | aOR | CI | aOR | CI | aOR | CI | aOR | CI |
| Age |  | 0.90 | 0.86-0.94 | 0.89 | 0.85-0.94 | 1.01 | 0.99-1.05 | 1.09 | 1.05-1.13 |
|  | female | 1 |  | 1 |  | 1 |  | 1 |  |
| Gender | male | 0.95 | 0.66-1.36 | 1.11 | 0.75-1.64 | 1.15 | 1.00-1.32 | 1.06 | 0.92-1.22 |
|  | low | 1 |  | 1 |  | 1 |  | 1 |  |
| SES score | medium | 3.72 | 2.51-5.53 | 3.39 | 2.27-5.06 | 1.39 | 1.13-1.72 | 1.18 | 0.95-1.49 |
|  | high | 4.19 | 2.64-6.67 | 4.13 | 2.55-6.70 | 1.08 | 0.87-1.35 | 0.93 | 0.72-1.20 |
| Migration background | none | 1 |  | 1 |  | 1 |  | 1 |  |
|  | one-sided | 0.72 | 0.42-1.25 | 1.15 | 0.65-2.03 | 1.13 | 0.88-1.48 | 1.12 | 0.86-1.45 |
|  | two-sided | 0.11 | 0.07-0.15 | 0.12 | 0.09-0.18 | 0.69 | 0.53-0.89 | 0.81 | 0.62-1.06 |
| Area of residence | east | 1 |  | 1 |  | 1 |  | 1 |  |
|  | west | 1.07 | 0.77-1.48 | 1.22 | 0.84-1.77 | 0.96 | 0.80-1.16 | 1.00 | 0.82-1.22 |
| Household size |  | 0.77 | 0.65-0.90 | 0.83 | 0.69-1.01 | 0.88 | 0.80-0.96 | 0.93 | 0.85-1.01 |
| Parents' marital status | single | 1 |  | 1 |  | 1 |  | 1 |  |
|  | married | 1.12 | 0.58-2.19 | 1.23 | 0.57-2.65 | 1.09 | 0.82-1.45 | 1.23 | 0.85-1.48 |
| Parents' smoking status | none | 1 |  | 1 |  | 1 |  | 1 |  |
|  | one parent | 0.84 | 0.58-1.23 | 1.03 | 0.67-1.58 | 1.12 | 0.92-1.36 | 1.13 | 0.94-1.38 |
|  | both parents | 0.92 | 0.55-1.53 | 0.95 | 0.55-1.65 | 1.05 | 0.84-1.30 | 1.17 | 0.95-1.44 |
| Physical Activity: active days per week | | 1.04 | 0.94-1.16 | 0.99 | 0.90-1.10 | 1.03 | 0.99-1.08 | 1.00 | 0.96-1.05 |
| Fast food consumption | | 0.87 | 0.82-0.92 | 0.86 | 0.80-0.93 | 0.96 | 0.92-1.01 | 1.05 | 0.98-1.10 |

*Table B3: Unadjusted regression models for J-examination use*

|  | | | J1 | | J2 | |
| --- | --- | --- | --- | --- | --- | --- |
|  |  |  | n = 3,537 | | n = 846 | |
|  |  |  | aOR | CI | aOR | CI |
| Age |  | | 1.34 | 1.26-1.42 | 2.34 | 1.69-3.24 |
| Gender | female | | 1 |  | 1 |  |
|  | male | | 1.09 | 0.90-1.34 | 0.79 | 0.53-1.18 |
| SES score | low | | 1 |  | 1 |  |
|  | medium | | 1.39 | 1.10-1.77 | 0.73 | 0.45-1.20 |
|  | high | | 1.13 | 0.85-1.50 | 0.64 | 0.36-1.15 |
| Migration background | none | | 1 |  | 1 |  |
|  | one-sided | | 0.91 | 0.68-1.22 | 0.87 | 0.45-1.67 |
|  | two-sided | | 0.76 | 0.57-1.03 | 0.80 | 0.44-1.45 |
| Area of residence | east | | 1 |  | 1 |  |
|  | west | | 0.85 | 0.67-1.08 | 0.72 | 0.48-1.11 |
| Household size |  | | 0.97 | 0.88-1.07 | 1.11 | 0.94-1.31 |
| Parents' marital status | single | | 1 |  | 1 |  |
|  | married | | 1.29 | 0.88-1.89 | 0.89 | 0.40-2.00 |
| Family cohesion |  | | 1.00 | 1.00-1.01 | 1.00 | 1.00-1.02 |
| Personal resources |  | | 1.00 | 1.00-1.01 | 1.00 | 0.99-1.01 |
| Self-efficacy |  | | 1.00 | 0.99-1.01 | 0.99 | 0.98-1.01 |
| Social support |  | | 1.00 | 1.00-1.01 | 1.00 | 0.99-1.02 |
| Parents' smoking status | none | | 1 |  | 1 |  |
|  | one parent | | 0.99 | 0.79-1.24 | 1.06 | 0.67-1.68 |
|  | both parents | | 0.93 | 0.70-1.23 | 0.75 | 0.42-1.33 |
| Physical Activity: active days per week | | | 0.97 | 0.92-1.02 | 1.05 | 0.95-1.16 |
| Fast food consumption | | | 0.98 | 0.93-1.02 | 0.98 | 0.89-1.06 |
| Ever consumed alcohol | | no | 1 |  | 1 |  |
|  |  | yes | 1.98 | 1.67-2.34 | 1.29 | 0.61-2.70 |
| Ever smoked | | no | 1 |  | 1 |  |
|  |  | yes | 1.34 | 0.89-2.02 | 1.02 | 0.59-1.75 |
