# Supplementary material for: The role of sociodemographic, psychosocial, and behavioral factors in the use of preventive healthcare services in children and adolescents: results of the KiGGS Wave 2 study
Source: BMC Pediatr. 2024 Feb 28;24:146. doi: 10.1186/s12887-024-04650-0 (PMC10900680; doi:10.1186/s12887-024-04650-0)
Supplement: Supplementary file 3 — Supplementary Material 3 [file 12887_2024_4650_MOESM3_ESM.docx]

*Table C1: Adjusted regression models for vaccination uptake*

|  | | Diphtheria | | Hepatitis B | | Hib | | Pertussis | | Polio | | Tetanus | |
| --- | --- | --- | --- | --- | --- | --- | --- | --- | --- | --- | --- | --- | --- |
|  |  | n = 3,238 | | n = 3,238 | | n = 3,238 | | n = 3,238 | | n = 3,238 | | n = 3,238 | |
|  |  | aOR | CI | aOR | CI | aOR | CI | aOR | CI | aOR | CI | aOR | CI |
| Age |  | 1.04 | 1.00-1.09 | 0.94 | 0.92-0.96 | 0.94 | 0.91-0.97 | 0.94 | 0.92-0.96 | 0.98 | 0.95-1.02 | 1.05 | 1.00-1.10 |
| Gender | female | 1 |  | 1 |  | 1 |  | 1 |  | 1 |  | 1 |  |
|  | male | 1.07 | 0.70-1.69 | 0.95 | 0.78-1.17 | 0.92 | 0.69-1.24 | 0.95 | 0.78-1.17 | 0.95 | 0.68-1.33 | 1.07 | 0.69-1.68 |
| SES score | low | 1 |  | 1 |  | 1 |  | 1 |  | 1 |  | 1 |  |
|  | medium | 2.52 | 1.51-4.22 | 1.16 | 0.87-1.53 | 1.2 | 0.81-1.75 | 1.16 | 0.87-1.53 | 1.72 | 1.13-2.60 | 2.91 | 1.71-4.97 |
|  | high | 1.74 | 0.90-3.47 | 1.08 | 0.76-1.54 | 0.9 | 0.56-1.47 | 1.08 | 0.76-1.54 | 1.26 | 0.74-2.18 | 2.2 | 1.09-4.64 |
| Migration background | none | 1 |  | 1 |  | 1 |  | 1 |  | 1 |  | 1 |  |
|  | one-sided | 3.48 | 1.18-16.85 | 1.15 | 0.81-1.68 | 2.24 | 1.15-5.03 | 1.15 | 0.81-1.68 | 2.20 | 1.05-5.59 | 4.77 | 1.31-38.23 |
|  | two-sided | 0.51 | 0.31-0.84 | 1.00 | 0.76-1.32 | 0.54 | 0.38-0.77 | 1.00 | 0.76-1.32 | 0.62 | 0.42-0.93 | 0.45 | 0.27-0.75 |
| Area of residence | east | 1 |  | 1 |  | 1 |  | 1 |  | 1 |  | 1 |  |
|  | west | 1.44 | 0.82-2.43 | 0.70 | 0.52-0.94 | 1.20 | 0.80-1.75 | 0.70 | 0.52-0.94 | 1.17 | 0.74-1.81 | 1.56 | 0.87-2.68 |
| Household size |  | 0.84 | 0.74-0.94 | 0.89 | 0.81-0.97 | 0.91 | 0.82-1.03 | 0.89 | 0.81-0.97 | 0.83 | 0.74-0.93 | 0.86 | 0.75-0.99 |
| Parents' marital status | single | 1 |  | 1 |  | 1 |  | 1 |  | 1 |  | 1 |  |
|  | married | 3.15 | 1.17-4.04 | 0.99 | 0.99-2.14 | 1.38 | 0.93-2.59 | 0.99 | 0.99-2.14 | 1.87 | 1.16-3.39 | 2.85 | 1.16-4.18 |
| Parents' smoking status | none | 1 |  | 1 |  | 1 |  | 1 |  | 1 |  | 1 |  |
|  | one parent | 1.09 | 0.69-1.75 | 1.19 | 0.93-1.52 | 0.97 | 0.70-1.36 | 1.19 | 0.93-1.52 | 1.05 | 0.72-1.55 | 1.18 | 0.73-1.94 |
|  | both parents | 3.08 | 1.45-7.62 | 1.73 | 1.24-2.47 | 1.47 | 0.92-2.45 | 1.73 | 1.24-2.47 | 1.57 | 0.93-2.79 | 3.57 | 1.60-9.48 |
| Pseudo-R^2^ | | 0.11 | | 0.10 | | 0.10 | | 0.08 | | 0.06 | | 0.12 | |
| aOR = adjusted odds ratio; CI = confidence interval; adjusted models included all listed variables adjusted for each other; age measured in years; household size measured in number of inhabitants; Pseudo-R^2^ = Cox & Snells Pseudo-R^2^ | | | | | | | | | | | | | |

*Table C2a: Adjusted regression models for U-examination use, U1–U4*

|  | | U1 | | U2 | | U3 | | U4 | |
| --- | --- | --- | --- | --- | --- | --- | --- | --- | --- |
|  |  | n = 14,017 | | n = 13,968 | | n = 13,964 | | n = 13,865 | |
|  |  | aOR | CI | aOR | CI | aOR | CI | aOR | CI |
| Age |  | 0.98 | 0.93-1.04 | 0.98 | 0.93-1.04 | 0.98 | 0.93-1.04 | 0.98 | 0.93-1.03 |
| Gender | Female(n/%) | 1 |  | 1 |  | 1 |  | 1 |  |
|  | male | 1.45 | 0.80-2.64 | 1.46 | 0.80-2.66 | 1.45 | 0.80-2.63 | 1.46 | 0.85-2.53 |
| SES score | low | 1 |  | 1 |  | 1 |  | 1 |  |
|  | medium | 2.92 | 1.60-5.35 | 2.92 | 1.60-5.35 | 2.91 | 1.59-5.34 | 2.60 | 1.52-4.44 |
|  | high | 2.39 | 1.06-5.39 | 2.43 | 1.07-5.48 | 2.44 | 1.08-5.52 | 2.48 | 1.10-5.56 |
| Migration background | none | 1 |  | 1 |  | 1 |  | 1 |  |
|  | one-sided | 1.39 | 0.35-5.59 | 1.39 | 0.35-5.60 | 1.39 | 0.34-5.59 | 0.41 | 0.11-1.56 |
|  | two-sided | 0.02 | 0.01-0.04 | 0.02 | 0.01-0.04 | 0.02 | 0.01-0.04 | 0.03 | 0.02-0.06 |
| Area of residence | east | 1 |  | 1 |  | 1 |  | 1 |  |
|  | west | 0.92 | 0.35-2.41 | 0.92 | 0.35-2.42 | 0.93 | 0.36-2.45 | 0.57 | 0.25-1.30 |
| Household size |  | 1.02 | 0.80-1.29 | 1.02 | 0.81-1.29 | 1.02 | 0.81-1.30 | 0.93 | 0.77-1.13 |
| Parents' marital status | single | 1 |  | 1 |  | 1 |  | 1 |  |
|  | married | 0.35 | 0.07-1.82 | 0.37 | 0.07-1.85 | 0.36 | 0.07-1.87 | 1.43 | 0.42-4.93 |
| Parents' smoking status | none | 1 |  | 1 |  | 1 |  | 1 |  |
|  | one parent | 1.31 | 0.64-2.67 | 1.32 | 0.65-2.69 | 1.32 | 0.65-2.72 | 1.48 | 0.76-2.88 |
|  | both parents | 2.25 | 0.78-6.50 | 2.29 | 0.79-6.59 | 2.28 | 0.79-6.57 | 1.98 | 0.75-5.25 |
| Pseudo-R^2^ | | 0.35 | | 0.36 | | 0.34 | | 0.30 | |
| aOR = adjusted odds ratio; CI = confidence interval; adjusted models included all listed variables adjusted for each other; age measured in years; household size measured in number of inhabitants; Pseudo-R^2^ = Cox & Snells Pseudo-R^2^ | | | | | | | | | |

*Table C2b: Adjusted regression models for U-examination use, U5–U7a*

|  | | U5 | | U6 | | U7 | | U7a | |
| --- | --- | --- | --- | --- | --- | --- | --- | --- | --- |
|  |  | n = 13,602 | | n = 13,266 | | n = 12,773 | | n = 11,679 | |
|  |  | aOR | CI | aOR | CI | aOR | CI | aOR | CI |
| Age |  | 0.96 | 0.91-1.01 | 0.92 | 0.87-0.97 | 0.95 | 0.89-1.00 | 0.84 | 0.82-0.86 |
| Gender | female | 1 |  | 1 |  | 1 |  | 1 |  |
|  | male | 1.29 | 0.78-2.13 | 1.16 | 0.69-1.94 | 1.13 | 0.73-1.76 | 1.03 | 0.85-1.25 |
| SES score | low | 1 |  | 1 |  | 1 |  | 1 |  |
|  | medium | 2.41 | 1.42-4.08 | 2.52 | 1.45-4.37 | 1.89 | 1.10-3.27 | 1.09 | 0.79-1.51 |
|  | high | 2.02 | 0.98-4.17 | 1.91 | 0.94-3.89 | 1.37 | 0.70-2.70 | 0.97 | 0.67-1.42 |
| Migration background | none | 1 |  | 1 |  | 1 |  | 1 |  |
|  | one-sided | 1.02 | 0.32-3.27 | 0.78 | 0.26-2.33 | 1.02 | 0.39-2.66 | 1.04 | 0.74-1.46 |
|  | two-sided | 0.04 | 0.02-0.08 | 0.05 | 0.03-0.08 | 0.07 | 0.05-0.10 | 0.49 | 0.37-0.64 |
| Area of residence | east | 1 |  | 1 |  | 1 |  | 1 |  |
|  | west | 1.13 | 0.61-2.12 | 1.61 | 0.87-2.97 | 1.55 | 0.89-2.69 | 1.24 | 1.01-1.53 |
| Household size |  | 0.96 | 0.80-1.16 | 0.97 | 0.78-1.19 | 0.95 | 0.79-1.16 | 0.91 | 0.81-1.02 |
| Parents' marital status | single | 1 |  | 1 |  | 1 |  | 1 |  |
|  | married | 1.33 | 0.40-4.39 | 1.40 | 0.44-4.50 | 1.26 | 0.58-2.76 | 1.24 | 0.79-1.94 |
| Parents' smoking status | none | 1 |  | 1 |  | 1 |  | 1 |  |
|  | one parent | 1.58 | 0.87-2.89 | 1.17 | 0.67-2.03 | 1.01 | 0.61-1.67 | 1.29 | 1.02-1.62 |
|  | both parents | 1.98 | 0.89-4.40 | 1.95 | 0.87-4.37 | 0.95 | 0.52-1.73 | 1.21 | 0.88-1.66 |
| Pseudo-R^2^ | | 0.27 | | 0.28 | | 0.21 | | 0.11 | |
| aOR = adjusted odds ratio; CI = confidence interval; adjusted models included all listed variables adjusted for each other; age measured in years; household size measured in number of inhabitants; Pseudo-R^2^ = Cox & Snells Pseudo-R^2^ | | | | | | | | | |

*Table C2c: Adjusted regression models for U-examination use, U8-U11*

|  | | U8 | | U9 | | U10 | | U11 | |
| --- | --- | --- | --- | --- | --- | --- | --- | --- | --- |
|  |  | n = 11,536 | | n = 10,358 | | n = 8,103 | | n = 6,383 | |
|  |  | aOR | CI | aOR | CI | aOR | CI | aOR | CI |
| Age |  | 0.93 | 0.89-0.98 | 0.92 | 0.86-0.97 | 1.03 | 1.00-1.07 | 1.12 | 1.08-1.17 |
|  | female | 1 |  | 1 |  | 1 |  | 1 |  |
| Gender | male | 0.70 | 0.47-1.03 | 0.86 | 0.56-1.31 | 1.12 | 0.96-1.31 | 1.05 | 0.90-1.22 |
|  | low | 1 |  | 1 |  | 1 |  | 1 |  |
| SES score | medium | 1.76 | 1.06-2.95 | 1.51 | 0.88-2.58 | 1.13 | 0.89-1.45 | 0.99 | 0.77-1.27 |
|  | high | 1.22 | 0.66-2.27 | 1.13 | 0.58-2.17 | 0.87 | 0.66-1.13 | 0.81 | 0.61-1.06 |
| Migration background | none | 1 |  | 1 |  | 1 |  | 1 |  |
|  | one-sided | 0.79 | 0.40-1.56 | 1.07 | 0.51-2.25 | 1.15 | 0.86-1.53 | 1.10 | 0.82-1.49 |
|  | two-sided | 0.12 | 0.08-0.18 | 0.14 | 0.09-0.22 | 0.83 | 0.63-1.08 | 0.94 | 0.71-1.26 |
| Area of residence | east | 1 |  | 1 |  | 1 |  | 1 |  |
|  | west | 1.69 | 1.10-2.61 | 1.60 | 0.95-2.71 | 0.97 | 0.79-1.19 | 1.00 | 0.81-1.22 |
| Household size |  | 0.96 | 0.79-1.16 | 1.01 | 0.81-1.25 | 0.90 | 0.83-0.99 | 0.96 | 0.87-1.05 |
| Parents' marital status | single | 1 |  | 1 |  | 1 |  | 1 |  |
|  | married | 1.08 | 0.54-2.14 | 0.93 | 0.39-2.22 | 1.19 | 0.85-1.67 | 1.05 | 0.74-1.49 |
| Parents' smoking status | none | 1 |  | 1 |  | 1 |  | 1 |  |
|  | one parent | 0.84 | 0.55-1.28 | 1.24 | 0.65-1.65 | 1.07 | 0.86-1.33 | 1.37 | 0.89-1.34 |
|  | both parents | 1.21 | 0.66-2.22 | 1.29 | 0.67-2.49 | 1.01 | 0.80-1.26 | 1.09 | 0.87-1.36 |
| Physical Activity: active days per week | | 0.96 | 0.86-1.07 | 0.94 | 0.85-1.03 | 1.06 | 1.01-1.11 | 1.04 | 1.00-1.09 |
| Fast food consumption | | 0.90 | 0.84-0.96 | 0.86 | 0.79-0.94 | 0.98 | 0.93-1.04 | 1.01 | 0.95-1.08 |
| Pseudo-R^2^ | | 0.17 | | 0.15 | | 0.16 | | 0.30 | |
| aOR = adjusted odds ratio; CI = confidence interval; adjusted models included all listed variables adjusted for each other; age measured in years; household size measured in number of inhabitants; Pseudo-R^2^ = Cox & Snells Pseudo-R^2^ | | | | | | | | | |

*Table C3: Adjusted regression models for J-examination use*

|  | | | J1 | | J2 | |
| --- | --- | --- | --- | --- | --- | --- |
|  |  |  | n = 3,537 | | n = 846 | |
|  |  |  | aOR | CI | aOR | CI |
| Age |  | | 1.36 | 1.25-1.48 | 2.73 | 1.86-4.02 |
| Gender | female | | 1 |  | 1 |  |
|  | male | | 0.97 | 0.76-1.24 | 0.71 | 0.43-1.17 |
| SES score | low | | 1 |  | 1 |  |
|  | medium | | 1.34 | 0.96-1.88 | 0.73 | 0.41-1.32 |
|  | high | | 1.06 | 0.71-1.57 | 0.45 | 0.21-0.98 |
| Migration background | none | | 1 |  | 1 |  |
|  | one-sided | | 1.02 | 0.72-1.44 | 0.67 | 0.29-1.54 |
|  | two-sided | | 1.05 | 0.73-1.52 | 0.82 | 0.40-1.67 |
| Area of residence | east | |  |  |  |  |
|  | west | | 0.84 | 0.63-1.12 | 0.79 | 0.49-1.29 |
| Household size |  | | 1.01 | 0.89-1.15 | 1.29 | 1.05-1.60 |
| Parents' marital status | single | | 1 |  | 1 |  |
|  | married | | 1.32 | 0.80-2.16 | 0.76 | 0.28-2.07 |
| Family cohesion |  | | 1.00 | 1.00-1.01 | 1.01 | 1.00-1.02 |
| Personal resources |  | | 1.01 | 1.00-1.02 | 1.01 | 0.98-1.02 |
| Self-efficacy |  | | 0.99 | 0.98-1.01 | 0.99 | 0.97-1.02 |
| Social support |  | | 1.01 | 1.00-1.01 | 1.01 | 0.99-1.03 |
| Parents' smoking status | none | | 1 |  | 1 |  |
|  | one parent | | 0.98 | 0.75-1.28 | 1.12 | 0.97-1.02 |
|  | both parents | | 1.01 | 0.71-1.45 | 0.8 | 0.70-1.78 |
| Physical Activity: active days per week | | | 0.97 | 0.92-1.03 | 1.01 | 0.91-1.12 |
| Fast food consumption | | | 0.97 | 0.92-1.03 | 0.97 | 0.88-1.06 |
| Ever consumed alcohol | | no | 1 |  | 1 |  |
|  |  | yes | 1.22 | 0.95-1.59 | 1.34 | 0.62-2.90 |
| Ever smoked | | no | 1 |  | 1 |  |
|  |  | yes | 1.03 | 0.60-1.77 | 1.03 | 0.54-1.99 |
| Pseudo-R^2^ | | | 0.10 | | 0.14 | |
| aOR = adjusted odds ratio; CI = confidence interval; adjusted models included all listed variables adjusted for each other; age measured in years; household size measured in number of inhabitants; Pseudo-R^2^ = Cox & Snells Pseudo-R^2^ | | | | | | |
